# A novel modification of Bosworth's technique to repair zone I Achilles tendon ruptures

**DOI:** 10.1007/s10195-012-0222-y

**Published:** 2013-01-11

**Authors:** Avadhanam Pavan Kumar, Raviprakash Shashikiran, Choulapalle Raghuram

**Affiliations:** 1 Chalmeda Anada Rao Institute of Medical Sciences, Bommakal, Karim nagar, Andhra Pradesh 505001 India; 2 Department of Orthopaedics, Chalmeda Anada Rao Institute of Medical Sciences, Bommakal, Karim nagar, Andhra Pradesh 505001 India

**Keywords:** Achilles tendon, Chronic rupture, Strip of the gastrocnemius aponeurosis, Zone I ruptures

## Abstract

**Background:**

Zone I ruptures of the Achilles tendon and chronic ruptures in zone II with a gap of more than 6 cm are difficult to treat. We describe a technique that is very well suited to this type of rupture.

**Materials and methods:**

Seventy-eight patients with chronic rupture of the Achilles tendon were operated on between January 1996 and December 2010. We used a modification of the Bosworth technique in which a strip of the gastrocnemius aponeurosis was taken, made into a tendon-like structure and passed through the calcaneum after making a drill hole; then it was sutured back to the proximal stump. The Leppilahti scoring system was used to evaluate these patients.

**Results:**

Sixty-two patients had excellent results, 8 had good results, 4 had fair results, 2 had poor results, and 2 were lost to follow-up at the end of 1 year. Nearly all patients resumed work at 6 months postoperatively, had normal walking and stair climbing, and regained normal dorsiflexion.

**Conclusion:**

Our technique is ideally suited to zone I ruptures (where no distal stump is available for repair) and ruptures in zone II where end-to-end repair is not possible.

## Introduction

Zone I of the Achilles tendon is the area from the calcaneal insertion to 2 cm above it. Ruptures and avulsions in this area are very common in patients with chronic tendinitis, peritendinitis, retrocalcaneal bursitis and a history of steroid injection at that level. Ruptures of the Achilles tendon in zone II (2–6 cm from the calcaneal insertion) that have been neglected for a long time have a distal stump that is not suitable for repair. Furthermore, neglected ruptures of the Achilles tendon pose a difficult problem, i.e., wide separation of the ruptured tendinous ends that cannot be repaired by end-to-end anastomosis even if the stump is available.

Several surgical procedures have been described with the objective of bridging this gap by grafting and thus restoring the continuity of the tendon. One or more Achilles tendon strips [1], the fascia lata [2] and tendons from the plantaris [3], peroneus brevis [4], flexor digitorum longus [5], flexor hallucis longus [6] or posterior tibial [7] tendons were the grafts utilized in these procedures. Synthetic materials, such as carbon fiber [8] or Dacron polyester [9], have also been used as scaffolding for the repair. All these studies described repair of the Achilles tendon in zone II. We could not identify articles on the management of zone I Achilles tendon ruptures and neglected ruptures with gaps of more than 6 cm. Most of the studies described the repair of the Achilles tendon with other small tendons. These small tendons do not have sufficient strength compared to the original Achilles tendon, and the surgical repair compromised the original function of the tendon.

The main objective of this study was to describe a novel technique of Achilles tendon repair suitable for ruptures in zone I and chronic ruptures in zone II where end-to-end repair is not possible. The outcomes were assessed with clinical and ultrasonographic evaluation.

## Materials and methods

From January 1996 through December 2010, 78 patients with long-standing rupture of the Achilles tendon were treated with the operative technique to be described (Table 1). All the patients gave informed consent prior to being included in the study. The study was approved by the local ethics committee and performed according to the Declaration of Helsinki. There were 48 men and 30 women with the ages ranging from 38 to 66 years. The duration of follow-up ranged from 6 months to 1 year. Forty-four patients had a history of previous injections of steroids around the Achilles tendon by other physicians for presumed tendinitis or retro-calcaneal bursitis. Fourteen patients had a history of severe trauma caused by sudden dorsiflexion of the foot. Twenty patients had a trivial traumas resulting in painful ankles and came without any history of steroid injection.

**Table 1:** Study overview

Characteristics	Value
Total no. of patients	78
Sex ratio M:F	48:30
Duration of follow-up	1 year
Zone I ruptures	72
Zone II ruptures	6
Mechanism of injury	
Steroid intake	44
Severe trauma	14
Trivial trauma	20

The duration of the symptoms ranged from 3 to 36 months. None of the cases were compound ruptures. All the patients had difficulty walking and pain around the ankle. Seventy of 78 patients were manual laborers; the other 8 patients were homemakers.

Because of the chronicity of the symptoms, none of the patients had ecchymosis, swelling and point tenderness, which are commonly associated with an acute rupture. There was only moderate swelling and edema around the posterior aspect of the ankle, but all patients had major weakness of active plantar flexion and a limp. In 55 patients, the defect in the Achilles tendon was visible or palpable. Usually there was some tenderness about the proximal and distal stumps of the ruptured tendon. The Thompson test was positive in all patients (that is, squeezing of the calf did not result in plantar flexion of the foot). Plain radiographs revealed Achilles tendon rupture by the gap between the tendon ends and presence of calcification in the distal portion of the proximal stump of the Achilles tendon.

All patients had undergone an ultrasound scan of the Achilles tendon to confirm the rupture. Seventy-two patients had a rupture in zone I (rupture within 2 cm of the calcaneal insertion). Six patients had a rupture in zone II (rupture 2 to 6 cm from the calcaneal insertion). The patients who received steroid injection had an avulsion of the tendon from the calcaneum. Intraoperatively, patients had necrosis of the tendon end, which was confirmed histologically. Calcification of the tendon along with necrosis was seen in a few patients.

### Operative technique

**Fig. 1:**
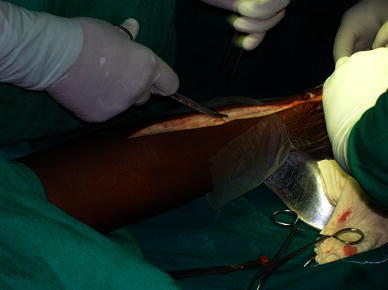
A long straight midline skin incision 10–12 cm below the knee joint. Isolation of the sural nerve and short saphenous vein is also seen

**Fig. 2:**
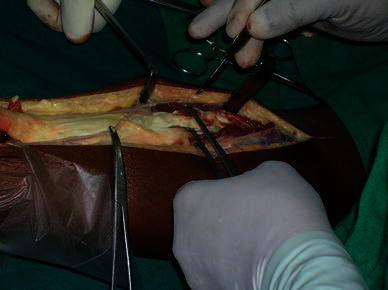
A 1.5–2-cm-wide strip cut and freed from the central portion of the raphe. Proximally the gastrocnemius muscle can be stripped from the apenurosis if more is required for repair

**Fig. 3:**
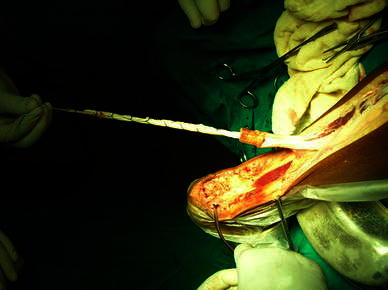
The processed strip of raphe passing through the stump for better anchorage and stability

**Fig. 4:**
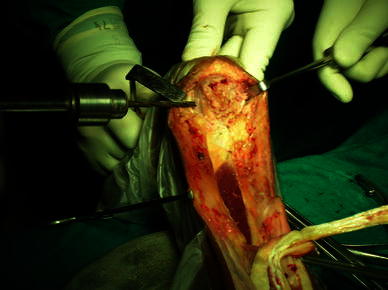
A transverse hole being made 1 cm below the Achilles tendon insertion in the calcaneum with a large-diameter drill bit

**Fig. 5:**
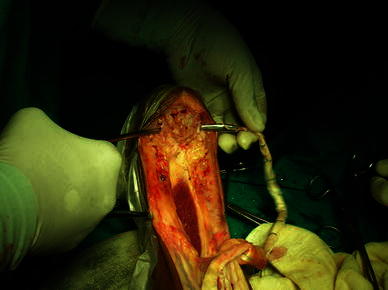
The processed raphe being passed from medial to lateral direction through the hole with the help of curved tendon tunneling forceps

**Fig. 6:**
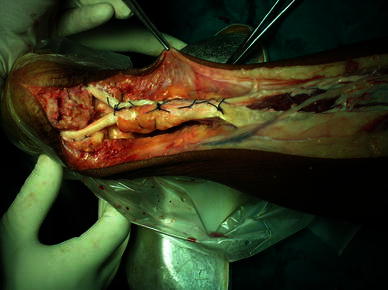
The processed raphe sutured back to the tendon while plantar flexing the ankle

The procedure was performed with the patient in semi-prone position and under general or spinal anesthesia with the use of tourniquet control. We used a straight midline incision starting proximally 10–12 cm below the knee joint (Fig. 1). This incision was continued distally and then gently curved laterally, distal to the insertion of the tendon. The length of the incision was 15–20 cm, sufficient for the exposure necessary to see the lesion completely and to execute the repair. After the skin incision, the sural nerve and short saphenous vein were secured and isolated. A 1.5–2-cm-wide strip was cut and freed from the central portion of the raphe (Fig. 2). Proximally the gastrocnemius muscle can be stripped from the apenurosis if more apenurosis is required for repair. The raphe should be taken from the tendon carefully as the tendon rotates for 90° from medial to lateral. The raphe was left attached just 1.5 cm proximal to the rupture site. The entire raphe strip was folded on itself with the smooth surface outside and sutured to make it into a long and thick structure simulating a tendon. The processed strip of raphe was passed through the stump for better anchorage and stability (Fig. 3). A transverse hole was made 1 cm below and behind the Achilles tendon insertion into the calcaneum with a large-diameter drill bit (Fig. 4). The processed raphe was passed in a medial to lateral direction through the hole with the help of curved tendon tunneling forceps, taking care that there was free movement of the tendon in bony tunnel; it was sutured back to the main tendon (Fig. 5). While suturing, a proper amount of tension in the tendon was maintained by plantar flexing the ankle (Fig. 6). The precautions taken during surgery were (1) making the skin incision up to the tendon without subcutaneous dissection and (2) tight continuous subcutaneous suturing to prevent wound dehiscence.

Postoperatively, a long, non-weight-bearing leg cast with the ankle in 20° plantar flexion and the knee in 30° flexion was applied for 4 weeks. Wound dressing was refreshed through a window over the cast 2 days after surgery. Three weeks after surgery, the sutures were removed. After 4 weeks, the knee was freed from the cast, and a short leg cast with the ankle in 20° plantar flexion was applied for 4 weeks. A walking short leg cast with the ankle plantigrade was applied for 4 more weeks and completely freed from casting after 3 months. After 3 months, gradual calf strengthening and stretching exercises were started along with weight bearing.

### Evaluation

The clinical outcome was assessed at 12-week, 6-month and 1-year follow-up visits using the clinical scoring method described by Leppilahti et al. [10]. The scoring included subjective factors such as pain, stiffness, muscle weakness and footwear restrictions; subjective outcomes as well as objective factors such as the active range of ankle motion and isokinetic calf muscle strength. The maximum number of points achievable was 100. The results were classified as excellent (≥90 points), good (75–89 points), fair (60–74 points) or poor (<60 points). The patients were asked to give answers to a nonvalidated subjective symptoms questionnaire. The patients were evaluated with an ultrasound scan at 6-month follow-up.

## Results

All patients were evaluated according to the Leppilahti scoring system (Table 2). Sixty-two patients had excellent results, eight had good results, and four had fair results. Two patients had poor results, and two were lost to follow-up after 1 year.

**Table 2:** Leppilahati scoring system

Clinical factor	Points	No. of patients at 6 months	At 1 year
Pain			
None	15	54	60
Mild, no limitations in recreational activities	10	20	15
Moderate, limitations in recreational, but not daily activities	5	1	1
Severe, limitations in recreational and daily activities	0	1	0
Stiffness			
None	15	40	57
Mild, occasional, no limitations in recreational activities	10	30	17
Moderate, limitations in recreational, but not daily activities	5	4	2
Severe, limitations in recreational and daily activities	0	2	0
Calf muscle weakness (subjective)			
None	15	50	70
Mild, occasional, no limitations in recreational activities	10	24	5
Moderate, limitations in recreational, but not daily activities	5	2	1
Severe, limitations in recreational and daily activities	0	0	0
Footwear restrictions			
None	10	60	62
Mild, most shoes tolerated	5	15	14
Moderate, unable to tolerate fashionable shoes, modified shoes tolerated	0	1	0
Active range of motion (ROM) difference between ankles			
Normal (<6°)	15	55	62
Mild (6°–10°)	10	14	7
Moderate (11°–15°)	5	6	5
Severe (>15°)	0	1	0
Subjective results			
Very satisfied	15	53	65
Satisfied with minor reservations	10	21	10
Satisfied with major reservation	5	2	1
Dissatisfied	0	0	0
Isokinetic muscle strength (score)			
Excellent	15	69	70
Good	10	3	4
Fair	5	4	2
Poor	0	0	0
Leppilahti score			
Excellent	90–100	58	62
Good	75–89	10	8
Fair	60–74	6	4
Poor	<60	2	2

**Fig. 7:**
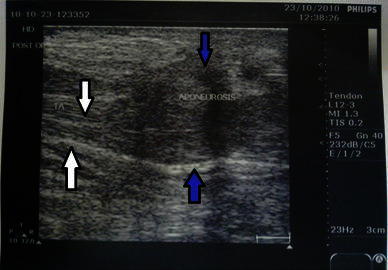
Ultrasound scan after 6 months showing continuation of fibers at the repaired site. The *blue arrows* show the repaired apenurosis, and the *white arrows* show intact tendon

All patients resumed work at 6 months postoperatively. Fifteen patients had mild pain at the end of 1 year at the ankle joint, but this did not hindering their daily or recreational activities. However, one patient had moderate pain that limited him from recreational activities but not from daily activities. Mild stiffness around the ankle joint was noted in 17 patients at the end of 1 year, but this stiffness did not limit their daily or recreational activities. Two patients complained of moderate stiffness. Nearly all patients had normal walking and stair climbing. There was significant improvement in the range of ankle motion postoperatively. There was an equal range of motion in both ankles in 62 out of 76 follow-up patients at the end of 1 year. Five patients were unable to raise their heels from the floor equally when on tiptoe because of other unrelated causes such as old age and obesity. Ultrasound scanning at 6-month postoperative follow-up showed increased thickness of the processed apenurosis, and delineation between the tendon end and processed apenurosis decreased (Fig. 7).

Complications in our series included scar hypertrophy (in 2 patients), superficial (in 2 patients), and deep infection (in 1 patient) and delayed wound healing (in 3 patients). Superficial infection was controlled with local antibiotics and regular dressings. Deep infection required a second debridement, and then the outcome was uneventful. In three patients, there was delayed healing, and it took nearly 1 month to heal completely with regular dressings. However, none of the patients had a re-rupture of the tendon.

## Discussion

A chronic rupture of the Achilles tendon causes difficulty and impairment of ankle plantar flexion. The tendon sheath often becomes thickened and adherent to the retracted ends of the tendon, and there is minimal repair tissue in the gap [7, 11, 12].

In neglected Achilles tendon ruptures, there is usually a gap between the tendon ends with retraction of the proximal stump, which results in shortening of the triceps surae muscle bellies and hence weakness of plantar flexion in the ankle. The tension that the muscle fiber can produce decreases as the fiber shortens until it becomes zero when the fiber is at approximately 60 % of its resting length. Most of the ruptures in our series had a gap of more than 6 cm (type 3 according to Meyerson’s classification and type 4 according to Kuwada’s classification [13]). Such cases cannot be treated conservatively, and surgical management is necessary to obtain normal range of ankle motion. The purpose of our study was to manage the Achilles tendon ruptures in zone I and chronic ruptures in zone II where end-to-end repair is not possible.

The etiology of Achilles tendon rupture remains unclear, but some investigations have proposed that chronic degenerative changes are a common cause based on the histological examination of the material obtained from the ruptured area during surgery [14, 15]. In our study, 64 patients sustained rupture of the Achilles tendon because of trivial trauma; of these, 44 patients had received local steroid injections for painful ankles. The remaining 20 patients with trivial ruptures had a history of long-term ankle pain. This suggests that most Achilles tendon ruptures are associated with chronic ankle pain (tendinitis and retrocalcaneal bursitis).

Balasubramaniam et al. [16] stated that injection of steroids into the tendon insertions caused necrosis at the site of injection and a delayed healing response. The anti-inflammatory and analgesic properties of corticosteroids may mask the symptoms of tendon damage, inducing individuals to maintain high levels of activity even when the tendon is damaged [17].

According to Langergran and Lindholm [18], the Achilles tendon is divided into three zones based on vascularity. Zone I is <3 cm from the calcaneal insertion, zone II 3–6 cm from the calcaneal insertion and zone III >6 cm from the calcaneal insertion. Most of the ruptures in our series were in zone I, with a negligible amount of distal stump present for end-to-end repair.

The techniques for repair or reconstruction of the neglected rupture can be broadly classified as end-to-end repair, Achilles tendon advancement or flap reconstruction, local tendon transfer and implantation (autograft, allograft or synthetic). According to Myerson, defects that are no more than 1–2 cm long can be managed with end-to-end repair and a posterior compartment fasciotomy. A gap up to 5 cm can be managed with V–Y lengthening, with or without a tendon transfer. When the space between tendon ends is >5 cm, it can be bridged with using a tendon transfer, alone or in combination with a V–Y advancement. However, when the rupture is in zone 1, end-to-end repair is not possible even though the gap between tendon ends is less than 2 cm. In such situations, our technique is ideal [19].

When the space between the tendon ends is ≥5 cm, bridging the gap with any other technique is tedious. With V–Y lengthening [20], gaps up to 5 cm can be repaired, but calf weakness is the main disadvantage. V–Y lengthening is not suitable when the gap is more than 6 cm.

Bosworth repaired Achilles tendon rupture by taking a wide strip of the proximal aponeurosis, which was woven through the proximal and distal tendon stumps. This repair needed some amount of distal stump for repair. This method is not ideally suited to ruptures in zone I. We modified the Bosworth technique by passing the apenurosis through the calcaneum instead of passing it through the distal stump and suturing it back to the main tendon, thus making it a good option for avulsions and ruptures in zone I [21].

White and Kraynick first reported a neglected rupture that was reconstructed with a peroneus brevis transfer to the calcaneus and reinforced with fascial strips. Teuffer described passing the peroneus brevis tendon through the calcaneus, across the repair site, and suturing it to the proximal Achilles tendon. Turco and Spinella augmented end-to-end repair of the Achilles tendon with a modification of Teuffer’s technique by passing the peroneus brevis through the distal tendon stump rather than the calcaneus [22, 23]. Two concerns, however, have been raised about this technique: first, the potential for eversion weakness. Secondly, because the tendon was placed distally in a lateral to medial direction, it did not duplicate the medial pull of the normal Achilles tendon.

Mann et al. [5] used the FDL to bridge the gap in chronic Achilles tendon ruptures. The FDL tendon was harvested in the midfoot, and the distal stump was sutured to the FHL. They also included a proximal fascial turndown flap in all cases and, when the length allowed, the proximal stump was reattached to the calcaneus with a pullout wire technique. This technique is not suitable when the gap between the ends is more than 5 cm.

Wapner et al. [6] reported on eight neglected ruptures that were reconstructed with the FHL transfer harvested from the midfoot. The main disadvantage of this technique is that the tendon is very thin in diameter, and it alone will not be adequate.

In our technique, we used aponeurosis of the gastronemius itself, thereby not needing tendon transfers, which would compromise the function of that tendon. As we were using the same tendon apenurosis, the strength remained balanced. Our technique would be ideal for a gap of more than 6 cm that needs tendon transfer with additional synthetic grafts.

Since this study is not prospective or randomized, the results cannot be considered conclusive. The best scientific conclusion can be derived if this method of repair is compared with other methods of repair. We had a limited follow-up period. Longer follow-up is needed to assess the long-term complications associated with our technique.

Our technique is ideally suited for chronic ruptures in zone I (where no distal stump is available for repair) and ruptures that have a large gap of more than 6 cm. This is accomplished with a single incision, with a good postoperative range of ankle motion and function.
